# Exceptional ancient DNA preservation and fibre remains of a Sasanian saltmine sheep mummy in Chehrābād, Iran

**DOI:** 10.1098/rsbl.2021.0222

**Published:** 2021-07-14

**Authors:** Conor Rossi, Gabriela Ruß-Popa, Valeria Mattiangeli, Fionnuala McDaid, Andrew J. Hare, Hossein Davoudi, Haeedeh Laleh, Zahra Lorzadeh, Roya Khazaeli, Homa Fathi, Matthew D. Teasdale, Abolfazl A'ali, Thomas Stöllner, Marjan Mashkour, Kevin G. Daly

**Affiliations:** ^1^ Smurfit Institute of Genetics, Trinity College Dublin, Dublin 2, D02 VF25, Ireland; ^2^ Austrian Academy of Sciences, Austrian Archaeological Institute, Archaeological Sciences, Hollandstraße 11-13, 1020 Vienna, Austria; ^3^ Central Laboratory, Bioarchaeology Laboratory, University of Tehran, 1417634934 Tehran, Iran; ^4^ Faculty of Humanities, Department of Archaeology, University of Tehran, 1417935840 Tehran, Iran; ^5^ McDonald Institute for Archaeological Research, Dept. of Archaeology, University of Cambridge, Cambridge CB2 3ER, UK; ^6^ Zanjan Cultural Heritage Centre, Archaeological Museum of Zanjan, Emaarate Zolfaghari, Taleghani St., Zanjan, Iran; ^7^ Research Department, Haus der Archäologien, Ruhr University Bochum, Institute for Archaeological Studies and Deutsches Bergbau-Museum Bochum, Am Bergbaumuseum 31, D-44791 Bochum, Germany; ^8^ Archéozoologie, Archéobotanique, Sociétés, Pratiques et Environnements (AASPE), Muséum national d'Histoire naturelle, Sorbonne Université, CNRS, CP 56, 55 rue Buffon, 75005 Paris, France

**Keywords:** ancient DNA, sheep, mummy

## Abstract

Mummified remains have long attracted interest as a potential source of ancient DNA. However, mummification is a rare process that requires an anhydrous environment to rapidly dehydrate and preserve tissue before complete decomposition occurs. We present the whole-genome sequences (3.94 X) of an approximately 1600-year-old naturally mummified sheep recovered from Chehrābād, a salt mine in northwestern Iran. Comparative analyses of published ancient sequences revealed the remarkable DNA integrity of this mummy. Hallmarks of postmortem damage, fragmentation and hydrolytic deamination are substantially reduced, likely owing to the high salinity of this taphonomic environment. Metagenomic analyses reflect the profound influence of high-salt content on decomposition; its microbial profile is predominated by halophilic archaea and bacteria, possibly contributing to the remarkable preservation of the sample. Applying population genomic analyses, we find clustering of this sheep with Southwest Asian modern breeds, suggesting ancestry continuity. Genotyping of a locus influencing the woolly phenotype showed the presence of an ancestral ‘hairy’ allele, consistent with hair fibre imaging. This, along with derived alleles associated with the fat-tail phenotype, provides genetic evidence that Sasanian-period Iranians maintained specialized sheep flocks for different uses, with the ‘hairy’, ‘fat-tailed’-genotyped sheep likely kept by the rural community of Chehrābād's miners.

## 1. Introduction

In 1993, a remarkably preserved human body dating to approximately 1700 years Before Present (BP) was discovered in the Douzlākh salt mine near Chehrābād village in the Zanjan Province of northwest Iran [1–3]. A total of eight ‘Salt Men’ have been identified at the mine [4,5], several retaining keratinous tissues such as skin, hair and both endo- and exoparasites, despite dating to the Achaemenid (550–330 BCE, 2500–2280 BP) and Sasanian (224–651 CE, approximately 1700–1300 BP) periods. The mine, also known as Chehrābād, was active in various periods, and its archaeological refilling layers represent an extraction history that ranged from the sixth century BCE to twentieth-century CE. In addition to the ‘Salt Men’, textiles, leather objects and animal remains have been discovered [6,7], likely preserved by the high salinity and low moisture content of the mine. Isotopic, genetic and lipid analyses have been reported for this material [1], and studies have been carried out to characterize genomic DNA survival [8]. These human and animal remains are examples of natural mummification—the spontaneous desiccation of soft tissue by a dry environment that rapidly dehydrates soft tissue before decay begins [9].

Mummification has been suggested as a mechanism that may sufficiently preserve keratinized tissue for ancient DNA (aDNA) sequencing [9]. The effects of age-related damage in aDNA are well documented and include base misincorporation at strand overhangs, fragmentation and low endogenous content [10]. Both deamination and depurination, associated with postmortem transition error and DNA fragmentation, respectively, require water as a substrate [10]. Ancient DNA from Chehrābād, a highly saline, anhydrous environment, presents an opportunity to investigate potential differences in nucleotide degradation resulting from this unusual taphonomic context.

**Figure 1.**
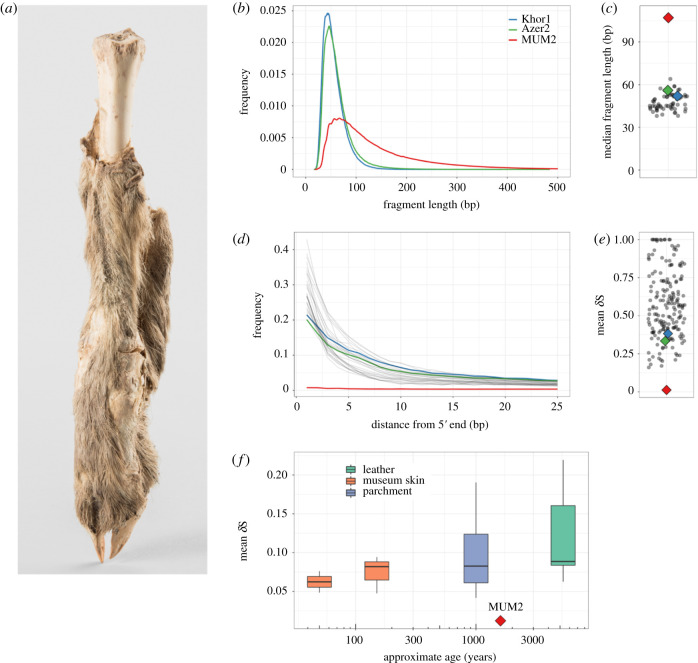
(*a*) Mummified sheep leg (4305) after cleaning. Photography: N. Tehrani. (*b*) Read length distributions of MUM2, Khor1 and Azer2, calculated from PE data. MUM2 shows a reduced rate of fragmentation. The median read length of MUM2 (107 bp) exceeds the median read length of Khor1 and Azer2 (52 bp and 56 bp, respectively). (*c*) Median read lengths of 61 published ancient Ovicaprid samples [11]. The median read length of MUM2 (107 bp; 90 bp among collapsed reads only) exceeds the longest among published ovicaprid genomes (64 bp). (*d*) Deamination patterns of MUM2, Khor1, Azer2 and other ancient ovicaprids for non UDG-treated libraries. Low levels of base misincorporation at the 5′ ends of reads were observed for MUM2 compared to Khor1 and Azer2. (*e*) Mean *δ*S of 182 published ancient bone samples [11,12]. The mean *δ*S of MUM2 (0.012) is unusual in its low levels of deamination. (*f*) Comparison of mean *δ*S of published ancient skins [13–15]. Lower damage rates are recorded compared to all samples, including some of approximately 50 years old.

In this study, we sequenced DNA from the approximately 1600-year-old (Sasanian period) mummified sheep leg 4305, recently discovered in a large mining gallery in the northwestern edge of the Douzlākh saltmine of Chehrābād by Iranian–German researchers during archaeological excavations (figure 1*a*) [2]. The specimen was likely deposited during refilling activities in the fourth–fifth centuries CE after the gallery's reopening in the Early Sasanian period (second–third centuries CE) and following its initial collapse between 405 and 380 BCE. The leg was possibly discarded during food preparation activities, as both sheep and goat were likely used as provisioning for Sasanian-period miners; equines may have been used as beasts of burden [16]. By this time, sheep were an established commodity for their meat and secondary products such as wool fibre, which was widespread by the fourth millennium BCE and showed regional specialization by the third millennium BCE [17].

We find unusual survival patterns of endogenous DNA given its distance from the equator, implying that exceptional preservation of nucleic acid integrity was afforded by the unique salt-rich environment. This enables characterization of the mummy skin metagenome and population genomic profiling of this sheep in the context of modern breeds. We also genotype 51 SNPs within the first intron of the platelet-derived growth factor D (*PDGFD*) that are highly differentiated between fat-tailed and thin-tailed breeds [18]. Finally we genotype the antisense *EIF2S2* retrogene insertion within the 3′ UTR of the *IRF2BP2* gene that influences the woolly phenotype and is derived relative to the ancestral coarse ‘hairy’ coat [19], in tandem with fibre analysis using scanning electron microscopy (SEM).

## 2. Material and methods

A sample of the mummified sheep skin (table 1, MUM2) from sheep leg 4305 was directly radiocarbon dated at the **^14^**CHRONO Centre (Queen's University Belfast). OxCal 4.3.2 [20] was used to calibrate its age (95.4% confidence interval) using [21].

**Table 1.** Summary information of samples sequenced in this study.

name	tissue	origin	period	age	endogenous DNA %	coverage (X)
MUM2	mummified skin	Chehrābād, Iran	Sasanian Empire period	399–539 cal CE^a^	31.01	3.94
Khor1	petrous bone	Nishapur, Iran	Sasanian–Islamic periods	600–1200 CE	58.44	0.04
Azer2	petrous bone	Tepe Hasanlu, Iran	Iron Age III	800–600 BCE	31.32	0.07

^a^directly dated.

Sample preparation, extraction and library preparation were performed in a dedicated aDNA laboratory in the Smurfit Institute of Genetics, Trinity College Dublin according to standard protocols (see electronic supplementary material). Sequencing of MUM2 and two Iranian sheep bone samples (Khor1 and Azer2) of approximately similar ages (table 1) for comparison was performed on Illumina MiSeq (50 bp SE) and HiSeq 2500 platforms (100 bp SE and 100 bp paired-end (PE)).

Sequencing reads were aligned to OviAri3.1 and filtered to produce bam files following standard aDNA sequencing pipelines (electronic supplementary material). Damage patterns were assessed using mapDamage2.0 [22].

Filtered reads not aligned to either sheep or human genomes were taxonomically assigned using the metagenomic classifier Kraken 2 [23]. Microbial sources were estimated using SourceTracker2 [24] with a custom metagenomic database [25–29] (electronic supplementary material). Bacterial species abundances were generated using MIDAS [30].

Mitochondrial sequences were produced using ANGSD [31] and a maximum-likelihood phylogenetic tree was generated using SeaView and PhyML [32–34] with the HKY85 substitution model, selected using jModelTest2 [35,36] and 100 bootstrap repeats.

A SNP dataset of modern breeds [37] was used to investigate genomic affinities (electronic supplementary material). LASER (v. 2.03) principal components analysis (PCA) [38], outgroup *f*_3_ statistics [39], TreeMix [40] and ADMIXTURE [41] analyses were completed (electronic supplementary material, table S5).

We investigated the woolly locus located on chromosome 25 [19]. Two modified OviAri3.1 assemblies were produced, one representing the ancestral ‘hairy’ phenotype, the other representing the ‘woolly’ phenotype (electronic supplementary material). Final bam files were visualized using IGV [42]. Hair fibres were examined using scanning electron and light microscopes at USTEM, TU Wien and Austrian Archaeological Institute, respectively. We assessed 51 SNPs in the *PDGFD* gene associated with the derived fat-tail phenotype [18], using the genotype calls of modern fat and thin-tailed breeds to define the derived allele [43,44]. As the average genome coverage was too low for accurate diploid genotype calls, we report base calls for both alleles.

## 3. Results and discussion

### (a) DNA preservation and metagenomics

The Chehrābād mummy sample (MUM2) was directly dated to the fifth–sixth century CE (2 sigma 1621–1481 cal BP, uncalibrated 1600 ± 30 BP; electronic supplementary material, figure S3). This aligns with the Sasanian Empire period of Iran, a time when the mine was in active use [1]. Initial DNA screening indicated high endogenous DNA for MUM2, and also the comparative Iranian sheep samples from relatively close time periods (table 1).

Sequencing of the Chehrābād mummy produced a 3.94 X genome after quality filtering (electronic supplementary material, table S1), in addition to the low coverage comparative genomes (0.0 4 X and 0.07 X). MUM2 differs from the two comparative sheep samples in displaying longer fragment lengths (median 107 bp versus 52 bp and 56 bp; figure 1*b*; collapsed reads-only 90 bp versus 50 bp and 55 bp) and substantially lower rates of deamination (figure 1*d*) (*δ*S, single-strand cytosine deamination probability, mean *δ*S = 0.012 versus 0.382 and 0.334). Contrasting previously published ancient ovicaprid data from Southwest Asia and Europe (electronic supplementary material, table S3), MUM2 falls outside the ranges of both median fragment length and mean *δ*S values (figure 1*c*,*e*), indicating remarkably low fragmentation and deamination of the Chehrābād sheep mummy genomic material given its latitude. Similar length distributions have been reported primarily from high latitude and permafrost environments [45–48]. A low level of thermal fluctuations may also contribute to DNA preservation [12], as comparable fragment lengths have been reported in a human sample from Wezmeh Cave, Iran [49].

Recent models of postmortem DNA fragmentation suggest rate-constant hydrolytic depurination over time [50], or age-independence, driven by environment-dependent biotic and abiotic factors [12]. The depurination rates of MUM2 are similar to the more-fragmented comparative samples (electronic supplementary material, figure S4), implying that other processes in the Chehrābād environment underlie the lower fragmentation rates. The highly alkaline, cool and anhydrous conditions may have contributed to the inhibition of cellular nucleases that would otherwise degrade and fragment endogenous DNA [9]. Postmortem DNA deamination via cytosine hydrolysis [51] is thought to be strongly correlated with age [52] and thermal age [12]. The substantially lower rates of deamination observed in MUM2 are likely owing to the scarcity of environmental free water, required for hydrolytic deamination. These results are consistent with Chehrābād providing a taphonomic environment conducive to genome preservation.

DNA preservation may also be influenced by its tissue-of-origin; for example, bone hydroxyapatite rather than keratin fractions is associated with smaller fragment size [53]. As hydrophobic keratinized tissue may provide resistance to environmental water [54], we compared MUM2 to published genomes of ancient skins (figure 1*f*) to determine if tissue providence was solely responsible for DNA preservation. The mean *δ*S of MUM2 falls outside the range of other ancient skin genomes, including twentieth-century CE goat skins [13] and leather recovered from the Tyrolean Iceman [14]. While this does not discount keratinized tissue being specifically enriched with longer DNA fragments, the Chehrābād sheep mummy appears to be singular in its DNA integrity among published skin samples.

Given the distinctive geochemical composition of Chehrābād, we examined if its salt-rich environment was reflected in the metagenomic profile of MUM2. Taxonomic assignment and abundance estimation assigned 57.13% of classified reads to the halophilic Class of Archaea *Halobacteria* (electronic supplementary material, table S2). Similarly, SourceTracker2 predicted that 0.4725–0.7458 of the microbial community originated from a salt-rich environment (table 2; electronic supplementary material, figure S5). A complementary analysis using MIDAS identified 76 unique bacterial species in the mummified sheep (electronic supplementary material, table S4). The most abundant species is the halophilic bacterium *Actinopolyspora halophila 58532,* accounting for approximately 29% of identified reads. This signal of a dominant halophilic microbial community is not replicated in comparison samples or controls (table 2; electronic supplementary material). Rapid colonization by saprophytic microbial communities, with key decomposers being ubiquitous across soil types, is typical for mammalian corpses postmortem [55]. The halophilic metagenome profile observed in the Chehrābād sheep mummy skin indicates that the typical decomposers may be less abundant in this alkaline, salt-rich setting, which may have contributed to soft tissue and molecular preservation.

**Table 2.** Predicted source proportion of metagenomic reads by SourceTracker2.

	MUM2 (species)	Azer2 (species)	Khor1 (species)	MUM2 (genus)	Azer2 (genus)	Khor1 (genus)
tissue decomposers	0.0212	0.1458	0.0386	0.0426	0.3524	0.3727
salt-rich	0.4725	0.0026	0.0036	0.7458	0.0145	0.0151
laboratory reagents	0.0003	0.0002	0.0002	N/A	N/A	N/A
sheep skin	0.0285	0.0850	0.0463	0.0829	0.3294	0.2244
soil	0.0009	0.0007	0.0046	0	0	0
unknown	0.4766	0.7657	0.9067	0.1287	0.3037	0.3878

### (b) Population genomics

We investigated how the Chehrābād sheep MUM2 relates to modern populations using a mitochondrial and autosomal variation. A 664 X mitochondrial genome of MUM2 falls within the C haplotype cluster in a maximum-likelihood phylogeny of modern sheep mitochondria (electronic supplementary material, figure S7). This clade is found at its highest frequency in southwest and east Asia [56,57], has been reported in ancient samples from Bronze Age Turkey [58] and is consistent with past and present-day patterns of mitochondrial diversity.

**Figure 2.**
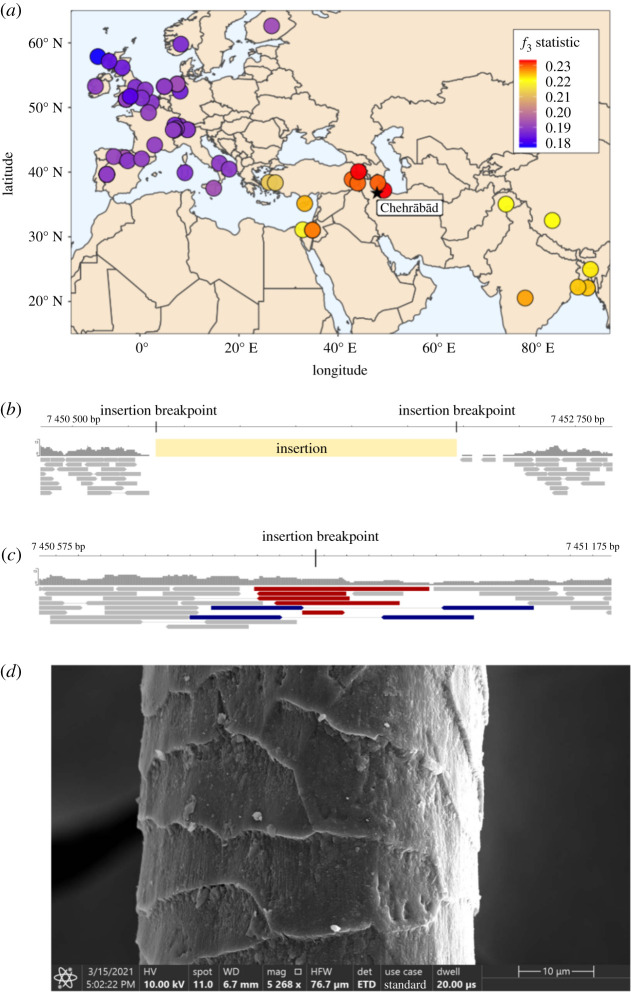
(*a*) Shared genetic drift between MUM2 and modern sheep populations. Higher *f*_3_ values, in red, indicate higher shared drift, relative to the outgroup Asiatic mouflon. Visualization of read coverage of filtered bam files at woolly locus, in assemblies with (*b*) and without (*c*) the insertion. Reads highlighted in red overlap the insertion breakpoint, blue indicates an inferred overlap of the insertion point by the straddling read pair. Highlighted reads map only to one assembly and do not align to the other. (*d*) SEM image of MUM2 hair fibre, displaying the mosaic scales typical of a sheep hair shaft. Image by A. Steiger-Thirsfeld and G. Ruß-Popa.

PCA from autosomal variation clusters MUM2 with modern southwest Asian breeds, using both global and Asian reference panels (electronic supplementary material, figure S8). *f*_3_ outgroup statistics show that MUM2 shares the most genetic drift with southwest Asian breeds, particularly those from Iran (figure 2*a*). ADMIXTURE and TreeMix analysis also confirmed the affinity of MUM2 with modern sheep breeds from southwest Asia (electronic supplementary material). Overall, there is genetic continuity between west Iranian sheep populations in Sassanid and modern time periods, although PCA using Ovine SNP50 genotypes of Asian breeds places MUM2 apart from sampled breeds (electronic supplementary material, figure S9), suggesting a degree of genetic flux during the past 1500–1600 years in Iranian sheep. This is consistent with evidence for genetic exchange across Asia prior to the development of modern breeds [59–61].

### (c) Fibre genotype and phenotype and fat-tail genotype

The derived ‘woolly’ coat phenotype is thought to be influenced by an approximately 1.5 kbp insertion of a *EIF2S2* retrogene into the *IRF2BP2* 3′ UTR, recessive to the ancestral allele associated with ‘hairy’ coat [19]. We exploited the length of the MUM2 DNA fragments to investigate this ‘woolly’ locus by searching for read pairs that either encompassed or overlapped the insertion breakpoint, indicative of a copy of the ‘hairy’ allele. No reads were found to overlap the diagnostic insertion breakpoints of the ‘woolly’ insert, which would indicate a copy of the ‘woolly’ allele (figure 2*b*). Five reads were found to uniquely map to the ‘hairy’ allele diagnostic position, with a further two read pairs inferred to overlap this breakpoint (figure 2*c*). We therefore infer this animal to be either homozygous or heterozygous for the dominant ‘hairy’ allele. In addition, SEM imaging of the mostly unpigmented mummified hair fibres revealed mosaic scales typical of sheep [62], with fine lines on the scale surface (figure 2*d*; electronic supplementary material), a characteristic of sheep hair fibres and particularly for mouflon and medium-wool breeds [63]. This may reflect MUM2 coming from a herd maintained for meat or milk production rather than wool, consistent with suggestions that ovicaprids were used as food for workers, and that sections of the mine were used as stables [1].

We also find evidence of a fat-tail associated allele (48/51 SNPs) (electronic supplementary material, table S6) at *PDGFD*, a gene likely controlling tail phenotype [18,44]. This observation, along with MUM2 sharing mitochondrial haplotype C with the majority of modern fat-tailed breeds [61], and the genomic affinity of MUM2 to modern fat-tailed breeds, although based on SNP-chip data, is intriguing. While we cannot determine the MUM2 tail phenotype directly, its genotype is similar to a medium-wool or hairy-coated fat-tail breed [1]. Hairy-coated sheep may have lower mortality rates, have higher birth weights, and be more robust than woolly coated [64], while fat-tail breeds are thought to be better adapted to arid environments [18]. If phenotypically similar to these sheep breeds, the flock represented by MUM2 could have provided a reliable meat and fat source for Chehrābād's miners. The faunal assemblage of the mine and paleoparasitological studies, although not very abundant, support the fact that sheep/goats were the most consumed animals by the miners [6,7,16,65].

Both woolly and fat-tailed sheep are depicted in the Early Bronze Age Mesopotamia but the spread of these phenotypes may have been uncoupled, and occurred via distinct processes [66,67]. Fat-tailed breeds were likely introduced from Southwest to East Asia in a period (700 BCE–1000 CE) broadly coinciding with the age of MUM2 [60]; the observed *PDGFD* genotype supports an ancient origin of this economically important trait. Wider aDNA analysis may elucidate when woolly and fat-tailed associated genotypes arose and how they may have influenced sheep breed development, which have their origins in fourth millennium BCE Mesopotamia [66,67]. Although the archaeozoological assemblages in the Iranian Plateau from the Antiquity and later Mediaeval periods are still limited, the diversity of the size of sheep bones is already an indication of the diversification of breeds in these periods [65,68]. Our results are consistent with MUM2 deriving from a herd used for meat and/or milk rather than wool production, and reflect sophisticated Sasanian-period husbandry practices and specialized sheep breeding.

## Data accessibility

Sequencing reads and mitochondrial sequences are available under European Nucleotide Archive accession PRJEB43881.

The data are provided in the electronic supplementary material [69].
